# Protection of taurine and granulocyte colony-stimulating factor against excitotoxicity induced by glutamate in primary cortical neurons

**DOI:** 10.1186/1423-0127-17-S1-S18

**Published:** 2010-08-24

**Authors:** Chunliu Pan, Amit Gupta, Howard Prentice, Jang-Yen Wu

**Affiliations:** 1 Department of Chemistry and Biochemistry, Florida Atlantic University, Boca Raton, FL 33431, USA; 2 College of Biomedical Science, Florida Atlantic University, Boca Raton, FL 33431, USA

## Abstract

**Abstracts:**

**Background**

Both taurine, an inhibitory neurotransmitter and granulocyte colony-stimulating factor (G-CSF), a growth factor, possess neuroprotective and neurotrophic properties in vitro. However, the mechanisms of their underlying neuroprotective effects are not fully understood.

**Methods**

In the present study, we investigated the potential protective benefits of taurine, G-CSF and the combination of taurine and G-CSF against excitotoxicity induced by glutamate in primary cortical neuronal cultures.

**Results**

25 mM taurine, 25 ng/ml G-CSF and the combination of 25 mM taurine and 25 ng/ml G-CSF showed a protective effect reaching 75%, 75% and 88%, respectively. Furthermore, taurine exerted its protective effect through down-regulation of expression of GRP 78, CHOP, Bim and caspase 12.

**Conclusion**

The results showed that all of these treatments, taurine, G-CSF and the combination of taurine and G-CSF, protected primary cortical neurons against excitotoxicity induced by glutamate. ER stress is suppressed by taurine after glutamate toxicity.

## Background

Taurine (2-aminoethanesulfonic acid), an inhibitory neurotransmitter, is present at high concentrations in many invertebrate and vertebrate systems [1-3]. Taurine has received much attention in the field of neuroprotection since the original experiments of Curtis and Watkins on the synaptic effects of inhibitory and excitatory amino acids [4,5]. Taurine is at a high level in the immature brain, serving as a trophic factor [6]. It has been thought to induce hyperpolarization, to inhibit firing of central neurons and to act as a modulator of synaptic activity in the brain [7-9]. The maintenance of the integrity of membranes, transmembrane Cl^-^ flux and intracellular calcium homeostasis are also important functions of taurine in the brain [10-13]. Taurine also acts as an osmoregulator and plays an antioxidant role [14-16]. In addition, it has been related to neuroprotection against multiple neurological diseases including Alzheimer’sdisease, Huntington’s disease and brain ischemia [17-19]. Moreover, taurine was found in neuronal systems to exert a protective function against toxicity induced by glutamate [20,21].

G-CSF is one of the few growth factors currently approved for clinical use for routine treatment of neutropenia [22]. It primarily stimulates proliferation, differentiation and maturation of cells committed to the neutrophilic granulocyte lineage through binding to the specific G-CSF receptor [23]. G-CSF also has been shown to have trophic effects on neuronal cells in vitro [24]. Moreover, G-CSF is an effective neuroprotectant in the treatment of a number of neurological diseases including stroke, Parkinson’s disease and Alzheimer’s disease [25-28]. In addition, apart from its protective role in neurons, G-CSF also dampens systemic inflammatory reactions, which may be of additional benefit in neurodegenerative conditions [29].

Although it is established that taurine and G-CSF have many beneficial effects under a variety of conditions of cell damage, the protective mechanisms are still unclear. We have recently demonstrated that taurine protects PC12 cells against ER stress induced by oxidative stress [30]. Here, we studied the protective effect of taurine, G-CSF and the combination of taurine and G-CSF against excitotoxicity induced by glutamate in rat primary neuronal cultures. We demonstrated that ER stress is also involved in the excitotoxicity induced by glutamate. Moreover, taurine protects primary neurons by suppressing ER stress induced by glutamate.

## Methods

### Materials

Basal medium-Eagle, fetal bovine serum, poly-D-lysine, taurine, Penicillin-Streptomycin and other chemicals were purchased from Sigma (St. Louis, MO, USA). Mouse anti-actin, rabbit anti-GRP78, rabbit anti-CHOP/GADD153, rabbit anti-caspase-12 antibodies and secondary mouse and rabbit antibodies were purchased from Santa Cruz Biotechnology (Santa Cruz, CA, USA). Rabbit anti-Bim antibody was purchased from Assay Designs (Ann Arbor, Michigan, USA). Adenosine 5’-triphosphate (ATP) Bioluminescent assay kit was purchased from Promega (Madison, WI, USA). RIPA buffer was purchased from Thermo Scientific (Rockford, IL, USA). Pregnant Sprague Dawley rats were purchased from Harlan (Indianapolis, IN) and housed in the animal care facility at Florida Atlantic University. The procedures for the care and use of rats, in accordance with the National Institutes of Health Guidelines for the Care and Use of Laboratory Animals, were approved by the Institutional Animal Care and Use Committee of Florida Atlantic University.

### Primary cortical neuronal cell culture

Primary cortical neuronal cell cultures were prepared using a previously described protocol [13]. Briefly, rat embryos at 17-18 days were removed and brains were isolated from the fetuses and kept in basal media Eagle (BME) supplemented with 2 mM glutamine, 26.8 mM glucose, and 20% heat-inactivated fetal bovine serum. This medium is referred to as growth medium-eagle (GME). The cortices then were dissociated by passing the tissue through a 14-G cannula. Cells were centrifuged at 200 g/min for 5 min at 25^o^C. The resulting pellet was resuspended in GME and plated on appropriate tissue culture plates precoated with 5 ug/ml of poly-D-lysine. Cells were maintained for 1 hour in a humidified incubator (37^o^C, 99% humidity and 5% CO2) before the incubation medium was replaced with serum-free neurobasalmedium (GIBCO) supplemented with B27 and 500 uM glutamine. The cultures were maintained in an incubator for 14 -18 days.

### Measurement of cell viability

Cells were measured by ATP assay. Neurons at 14 days in vitro were preincubated with 25 mM taurine for 1 hour. Then the neurons were treated with 100 uM glutamate for 4 hours. ATP solution was added to each well and cells were incubated for 10 minutes, after which levels of ATP were quantified in a luciferase reaction. The luminescent intensity was measured using a luminometer (SpectraMax, Molecular Devices) after transferring the lysate to a standard opaque walled multi-well plate.

### Western blot analysis

Primary cortical neuron cultures were lysed in RIPA buffer (25 mM Tris-HCl pH 7.6, 150 mM NaCl, 1% NP-40, 1% sodium deoxycholate, 0.1% SDS) containing 1% (v/v) mammalian protease inhibitor cocktail from Sigma and separated on a SDS-PAGE. After proteins were transferred to a nitrocellulose membrane, the membrane was then blocked in blocking buffer (20 mM Tris-HCl, 150 mM NaCl, 0.1% Tween-20, 5% milk) for 1.5 hours at room temperature. After blocking, the corresponding primary antibody was incubated for one hour, followed by one hour incubation with the corresponding HRP-conjugated secondary antibody at room temperature. Extensive washes with blocking buffer were performed between each step. The protein immuno-complex was visualized using ECL detection reagents.

### Statistical analysis

All data shown were expressed as the mean ± SEM. The Student’s t-test or one-way ANOVA was used to compare means between groups. Differences of P<0.05 were considered statistically significant.

## Results

### Dose-dependent glutamate toxicity in primary neuron cultures

**Figure 1:**
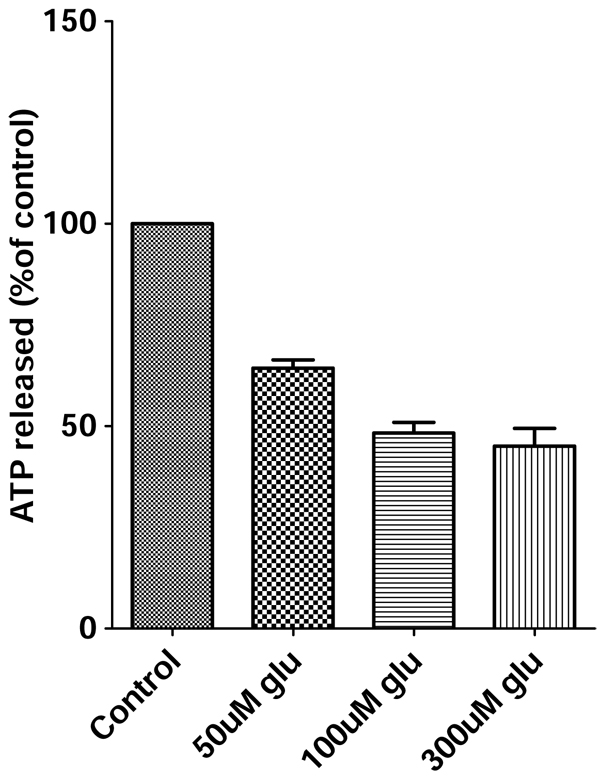
**Effect of glutamate on cell viablility - A dose-dependent study.** Primary cortical neurons were exposed to 50 uM, 100 uM and 300 uM glutamate for 4 hours and cell viability was measured by ATP assay.

Excessive levels of the neurotransmitter glutamate trigger excitotoxic processes in neurons that result in cell death [31].To identify the excitotoxic dose range of glutamate, rat cortical neurons were treated for 4 hours with 50, 100, 200 or 300 uM glutamate respectively. The results are shown in Fig. 1. We found that glutamate treatment caused a dose-dependent increase in neuronal apoptotic processes. There was approximately 50% survival of cortical neurons with 100 uM glutamate treatment for 4 hours (Fig. 1, lane 3). 100 uM glutamate was chosen as an optimal concentration to induce the excitotoxicity.

### Protective effects of taurine against glutamate toxicity in primary neuron cultures

**Figure 2:**
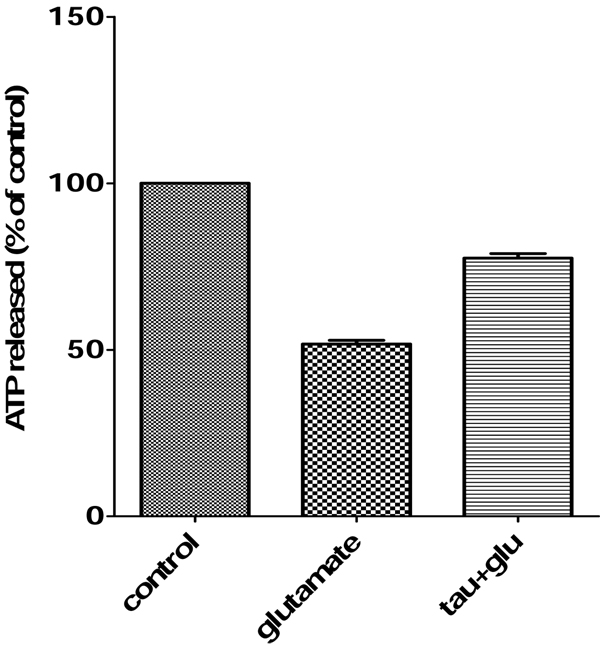
**Neuroprotective effect of taurine against glutamate-induced excitotoxicity measured by ATP assay**. Primary cortical neurons were treated with 25 mM taurine for 1 hour before exposure to 100uM glutamate for 4 hours and cell viability was measured by ATP assay.

Previously, we found that 25 mM taurine resulted in the optimal neuroprotection against glutamate induced excitotoxicity [32]. For this reason, we selected the 25 mM taurine concentration for testing cell viability using the ATP assay. For testing the protective effect of 25 mM taurine against glutamate in cortical neurons, cells were seeded in 96-well plates and treated with or without 25 mM taurine for 1 hour followed by 100 uM glutamate exposure for 4 hours. The cell survival results are shown in Fig. 2. The treatment of 25 mM taurine increased the cell survival by 75% compared to the condition with 100 uM glutamate treatment.

### Protection of G-CSF against glutamate toxicity in primary neuronal cultures

**Figure 3:**
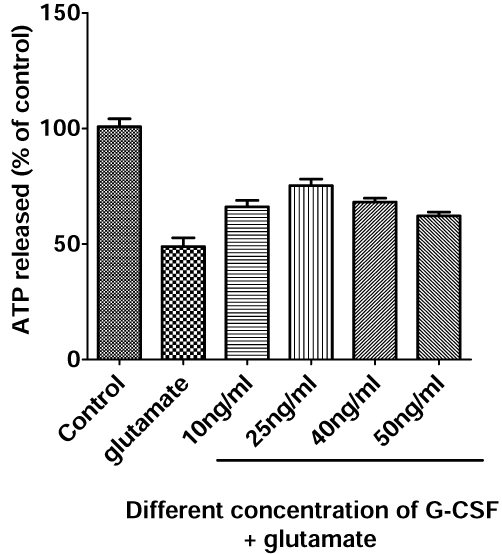
**Neuroprotective effect of G-CSF against glutamate-induced excitotoxicity - A dose-dependent study.** Primary cortical neurons were preincubated with 10, 25, 40 and 50ng/ml G-CSF for 1 hour, then exposed to 100uM glutamate for 4 hours. Cell viability was measured by ATP assay.

G-CSF has been widely investigated in terms of protection of neurons in stroke, as shown in numerous papers [25,26,33-36]. Glutamate has been shown to play a key role in the pathogenesis of stroke [37]. However, there has been little research on the protective function of G-CSF in glutamate induced excitotoxicity in vitro. G-CSF was previously shown to exhibit a protective effect in cerebellar granule cells exposed to glutamate toxicity [25]. In the current study, we demonstrated the protective function of G-CSF at a range of concentrations from 10 to 40 ng/ml against excitotoxicity induced by glutamate in primary neuronal cultures (Fig. 3). G-CSF treatment resulted in an enhanced cell survival at several concentrations, with the highest protection of 75% occurring at 25 ng/ml.

### The protective effect of the combination of taurine and G-CSF in primary neuronal cultures

**Figure 4:**
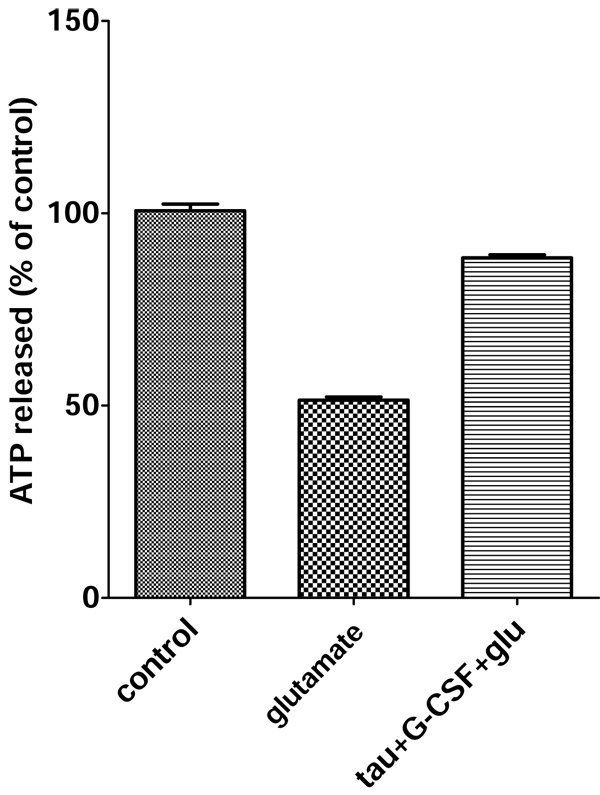
**Neuroprotective effect of the combination of taurine and G-CSF against glutamate-induced excitotoxicity**. Primary cortical neurons were preincubated with 25 mM taurine and 25 ng/ml G-CSF for 1 hour, and then exposed to 100 uM glutamate for 4 hours. Cell survival was measured by ATP assay.

To test whether the combination of taurine and G-CSF promotes protection against glutamate induced toxicity, we treated primary neurons with 25 mM taurine plus 25 ng/ml G-CSF for 1 hour, followed by glutamate treatment for 4 hours. The results are shown in Fig. 4. The combination of taurine and G-CSF increased the protective effect against glutamate toxicity to 88% cell survival compared to 75% cell survival from taurine or G-CSF treatment alone.

### Taurine protects neurons against glutamate excitotoxicity by suppressing the expression of GRP78, CHOP, Caspase-12 and Bim

**Figure 5:**
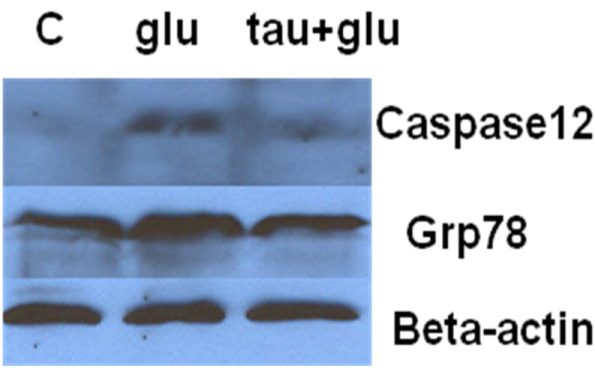
**Effect of taurine on glutamate-induced elevated expression of GRP78 and Caspase-12 by Western blot analysis**. Primary cortical neurons were preincubated with 25 mM taurine for 1 hour, and then exposed to 100 uM glutamate for 4 hours. Expression of GRP78 protein and caspase 12 was analyzed by Western blot assay. Beta-actin was included to show equal loading. **C**: Control; **glu**: Glutamate-treated group; **tau+ glu**: Same as **glu** group except pretreated with taurine.

**Figure 6:**
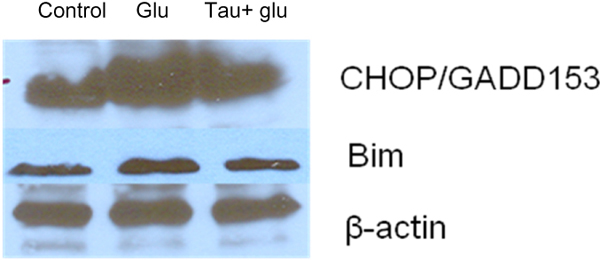
**Effect of taurine on glutamate-induced elevated expression of CHOP and Bim by Western blot analysis**. Primary cortical neurons were treated the same as described in Figure 5 except that the expression of CHOP and Bim was analyzed by Western blot assay.

To investigate if ER stress can be induced by glutamate and then suppressed by taurine, specific ER stress effector proteins were analyzed by western blot. Glucose regulated protein-78 (GRP78) is an ER-associated chaperone, which facilitates protein folding in ER [38]. The expression of GRP78 protein was up-regulated in primary neurons after treatment with 100 uM glutamate for 4 hours. However, taurine restored the level of GRP78 to control levels, as shown in Fig. 5. C/EBP homologous protein (CHOP), also known as growth arrest and DNA damage inducible protein 153 (GADD153), is an important ER stress marker [39]. Fig. 6 shows that the expression of CHOP was up-regulated by glutamate. Taurine treatment restored CHOP expression to the control level (Fig. 6). Both Caspase-12 and Bim play an essential role in the progression of programmed cell death during the proapoptotic phase of the ER stress response [40,41]. Taurine reversed the induction of Caspase-12 and Bim caused by glutamate in primary neurons, as shown in Fig. 5 and Fig. 6.

## Discussion

In the present study, we have demonstrated the potent protection by taurine and by G-CSF in an *in vitro* model of primary cortical neuronal cell death induced by glutamate. Taurine and G-CSF protected primary cortical neurons against glutamate-induced neurotoxicity as determined by measuring cell viability using the ATP assay. On the other hand, we found that the combination of taurine and G-CSF gave a synergistic enhancement of protection against glutamate in primary cortical neurons. We have further shown that the suppression of ER stress is an essential underlying mechanism for taurine-induced neuroprotection. Our investigation of the intracellular mechanisms downstream of ER stress demonstrated a reversal by taurine of glutamate-induced increases in GRP78, CHOP, Caspase-12 and Bim levels.

A previous paper reported that taurine and basic fibroblast growth factor (bFGF) in combination gave an enhanced neuroprotection in granule neurons against glutamate induced excitotoxicity [42]. They showed that neuroprotection was obtained only through the combined action of taurine and bFGF in a cerebellar granule neuron rich culture, but not by these factors alone. Therefore, they believed that taurine can augment bFGF function under certain conditions. Here, we demonstrated that taurine or G-CSF administrated alone showed a neuroprotective effect. Furthermore, an enhanced protection against glutamate was also observed with a combination of taurine and G-CSF. The clinical application of taurine was investigated and found to be effective in studies as early as 1974 when it was applied to treatment for refractory epilepsy [43]. Both taurine and G-CSF have been shown to be potential drugs for ischemia or stroke in clinical applications [44,45]. Since the combination of taurine and G-CSF have synergistic neuroprotective effects against glutamate excitotoxicity, as demonstrated in this paper, this strongly suggests that the combination of taurine and G-CSF may be more effective than the individual agents in treatment of neurological diseases, such as stroke.

Many neurological disorders such as Alzheimer’s disease, stroke and Parkinson’s disease have been linked to the overactivation of glutamatergic transmission and excitotoxicity as a common pathway of neuronal injury [46-48]. Previous studies have also shown that ER stress is induced in neurons by glutamate toxicity [49,50]. Recently, kainic acid (KA), a non-NMDA glutamate receptor agonist, was found to cause the disintegration of the ER membrane in hippocampal neurons and to cause ER stress [51]. In this study, we demonstrated glutamate induced ER stress associated with the up-regulation of the proteins GRP78, CHOP, Bim and caspase-12.

Although taurine has been investigated and applied to treat many diseases, the protective mechanism is still not fully understood. We have already demonstrated that ER stress induced by H_2_O_2_ in PC12 cells was prevented by taurine treatment [30]. In the present study, our results show that taurine reduces the ER stress induced by glutamate in primary neuronal cultures.

## Conclusion

In the present study, we demonstrated that both taurine and G-CSF protect primary cortical neurons against glutamate-induced cell death. Interestingly, we found that the combination of taurine and G-CSF results in an enhanced protective effect. Because both taurine and G-CSF are neuroprotective agents that are approved for clinical use, the combined administration of these two factors may constitute a viable therapy with potentially enhanced therapeutic efficacy. Moreover, taurine suppressed the ER stress induced by glutamate. Further investigation will be performed to examine the specific pathway responsible for ER stress induced by glutamate and to identify molecular targets in the ER stress pathway that are specifically inhibited by taurine, G-CSF and their combination.

## Competing interests

The authors declare that they have no competing interests.

## References

[B1] Huxtable RJ The physiological actions of taurine Physiol. Rev 1992 72 101 163 1731369 10.1152/physrev.1992.72.1.101

[B2] Sturman JA Taurine in development Physiol. Rev 1993 73 119 147 8419963 10.1152/physrev.1993.73.1.119

[B3] Huxtable RJ Taurine in the central nervous system and the mammalian actions of taurine Prog. Neurobiol 1989 32 471 533 10.1016/0301-0082(89)90019-1 2664881

[B4] Curtis DR Watkins JC The excitation and depression of spinal neurones by structurally related amino acids J. Neurochem 1960 6 117 141 10.1111/j.1471-4159.1960.tb13458.x 13718948

[B5] Curtis DR Watkins JC Analogues of glutamic and gamma-aminobutyric acids having potent action on mammalian neurons Nature (Lond) 1961 191 1010 10.1038/1911010a0 13718947

[B6] Palackal T Kujawa M Moretz RC Neuringer M Sturman JA Laminar analysis of the number of neurons, astrocytes, oligodendrocytes amd microglia in the visual cortex (area 17) of 3-month-old rhesus monkeys fed a human infant soy-protein formula with or without taurine supplementation from birth Dev. Neurosci 1991 13 20 33 10.1159/000112137 2055169

[B7] Kuriyama K Taurine as a neuromodulator Fed. Proc 1980 39 2680 2684 6105096

[B8] Oja SS Saransaari P Taurine as osmoregulator and neuromodulator in the brain Metab Brain Dis 1996 11 153 164 10.1007/BF02069502 8776717

[B9] Saransaari P Oja SS Release of GABA and taurine from brain slices Prog. Neurobiol 1992 38 455 482 10.1016/0301-0082(92)90046-H 1589578

[B10] Moran J Salazar P Pasantes-Morales H Effect of tocopherol and taurine on membrane fluidity of retinal rod outer segments Exp Eye Res 1987 45 769 776 10.1016/S0014-4835(87)80094-5 3428399

[B11] Okamoto K Kimura H Sakai Y Taurine-induced increase of the Cl-conductance of cerebellar Purkinje cell dendrites in vitro Brain Res 1983 259 319 323 10.1016/0006-8993(83)91266-0 6297677

[B12] Satoh H Sperelakis N Review of some actions of taurine on ion channels of cardiac muscle cells and others Gen. Pharmacol 1998 30 451 463 10.1016/S0306-3623(97)00309-1 9522160

[B13] Chen WQ Jin H Nguyen M Carr J Lee YJ Hsu CC Faiman MD Schloss JV Wu JY Role of taurine in regulation of intracellular calcium level and neuroprotective function in cultured neurons J Neurosci Res 2001 66 612 619 10.1002/jnr.10027 11746381

[B14] Wade JV Olson JP Samson FE Nelson SR Pazdernik TL A possible role for taurine in osmoregulation within the brain J. Neurochem 1988 51 740 745 10.1111/j.1471-4159.1988.tb01807.x 3411323

[B15] Militante J Lombardini JB Age-related retinal degeneration in animal models of aging: Possible involvement of taurine deficiency and oxidative stress Neurochem Res 2004 29 151 160 10.1023/B:NERE.0000010444.97959.1b 14992274

[B16] Balkan J Kanbagli O Hatipoglu A Kucuk M Cevikbas U Toker G Uysal M Improving effect of dietary taurine supplementation on the oxidative stress and lipid levels in the plasma, liver and aorta of rabbits fed on a high-cholesterol diet Biosci Biotechnol Biochem 2002 66 1755 1758 10.1271/bbb.66.1755 12353642

[B17] Takatani T Takahashi K Uozumi Y Shikata E Yamamoto Y Ito T Matsuda T Schaffer SW Fujio Y Azuma J Taurine inhibits apoptosis by preventing formation of the Apaf-1-caspase-9 apoptosome Am J Physiol Cell Physiol 2004 287 C949 C953 10.1152/ajpcell.00042.2004 15253891

[B18] Paula-Lima AC Felice GF Brito-Moreira J Ferreira TS Activation of GABAA receptors by taurine and muscimol blocks the neurotoxicity of beta-amyloid in rat hippocampal and cortical neurons Neuropharmacology 2005 49 1140 1148 10.1016/j.neuropharm.2005.06.015 16150468

[B19] Tadros GM Khalifa EA Abdel-Naim BA Arafa Huntingtons Neuroprotective effect of taurine in 3-nitropropionic acid-induced experimental animal model of Huntington's disease phenotype Pharmacol Biochem Behav 2005 82 574 582 10.1016/j.pbb.2005.10.018 16337998

[B20] Ward R Cirkovic-Vellichovia T Ledeque F Tirizitis G Dubars G Datla K Dexter D Heushling P Crichton R Neuroprotection by taurine and taurine analogues Adv Exp Med Biol 2006 583 299 306 full_text 17153614 10.1007/978-0-387-33504-9_33

[B21] Wu JY Wu H Jin Y Wei J Sha D Prentice H Lee HH Lin CH Lee YH Yang LL Mechanism of Neuroprotective Function of Taurine Adv Exp Med Biol 2009 643 169 179 full_text 19239147 10.1007/978-0-387-75681-3_17

[B22] Metcalf D The colony stimulating factors discovery, development, and clinical applications Cancer 1990 65 2185 95 10.1002/1097-0142(19900515)65:10<2185::AID-CNCR2820651005>3.0.CO;2-4 2189549

[B23] Hartung T Anti-inflammatory effects of granulocyte colony-stimulating factor Curr Opin Hematol 1998 5 221 225 10.1097/00062752-199805000-00013 9664164

[B24] Konishi Y Chui DH Hirose H Kunishita T Tabira T Trophic effects of erythropoeitin and other hematopoietic factors on central cholinergic neurons in vitro and in vivo Brain Res 1993 609 29 35 10.1016/0006-8993(93)90850-M 7685231

[B25] Schabitz WR Kollmar R Schwaninger M Juettler E Bardutzky J Scholzke MN Sommer C Schwab S Neuroprotective effect of granulocyte colonystimulating factor after focal cerebral ischemia Stroke 2003 34 745 751 10.1161/01.STR.0000057814.70180.17 12624302

[B26] Solaroglu I Tsubokawa T Cahill J Zhang JH Anti-apoptotic effect of granulocyte-colony stimulating factor after focal cerebral ischemia in the rat Neuroscience 2006 143 965 974 10.1016/j.neuroscience.2006.09.014 17084035 PMC1820637

[B27] Meuer K Pitzer C Teismann P Krüger C Göricke B Laage R Lingor P Peters K Schlachetzki JC Kobayashi K Dietz GP Weber D Ferger B Schäbitz WR Bach A Schulz JB Bähr M Schneider A Weishaupt JH Granulocyte-colony stimulating factor is neuroprotective in a model of Parkinson’s disease J Neurochem 2006 97 675 686 10.1111/j.1471-4159.2006.03727.x 16573658

[B28] Tsai KJ Tsai YC Shen CK G-CSF rescues the memory impairment of animal models of Alzheimer’s disease J Exp Med 2007 204 1273 1280 10.1084/jem.20062481 17517969 PMC2118601

[B29] Hartung T Anti-inflammatory effects of granulocyte colony-stimulating factor Curr Opin Hematol 1998 5 221 225 10.1097/00062752-199805000-00013 9664164

[B30] Pan C Giraldo GS Prentice H Wu JY Taurine protection of PC12 cells against endoplasmic reticulum stress induced by oxidative stress J Biomed Sci 2010 17 Suppl 1 S17 10.1186/1423-0127-17-S1-S17 PMC2994405 20804591

[B31] Meldrum B Garthwaite J Excitatory amino acid neurotoxicity and neurodegenerative disease Trends Pharmacol Sci 1990 11 379 87 10.1016/0165-6147(90)90184-A 2238094

[B32] Leon R Wu H Jin Y Wei J Buddhala C Prentice H Wu J-Y Protective function of taurine in glutamate-induced apoptosis in cultured neurons J Neurosci Res 2009 87 1185 1194 10.1002/jnr.21926 18951478

[B33] Hasselblatt M Jeibmann A Riesmeier B Maintz D Schaitz W-R Granulocyte-colony stimulating factor (G-CSF) and G-CSF receptor expression in human ischemic stroke Acta Neuropathol 2007 113 45 51 10.1007/s00401-006-0152-y 17047971

[B34] Lee S-T Chu K Jung K-H Ko S-Y Kim E-H Sinn D-I Lee Y-S Lo EH Kim M Roh J-K Granulocyte colony-stimulating factor enhances angiogenesis after focal cerebral ischemia Brain Res 2005 1058 120 128 10.1016/j.brainres.2005.07.076 16150422

[B35] Yataa K Matchettc AG Tsubokawaa T Tanga J Kanamarud K Zhang HJ Granulocyte-colony stimulating factor inhibits apoptotic neuron loss after neonatal hypoxia–ischemia in rats Brain Res 2007 1145 227 238 10.1016/j.brainres.2007.01.144 17359943 PMC1888563

[B36] Chen WF Jean YH Sung CS Wu GJ Huang SY Ho JT Su TM Wen ZH Intrathecally injected ranulocyte colony-stimulating factor produced europrotective effects in spinal cord ischemia via the mitogen-activated protein kinase and Akt pathways Neuroscience 2008 153 31 43 10.1016/j.neuroscience.2008.01.062 18358629

[B37] Aliprandi A Longoni M Stanzani L Tremolizzo L Vaccaro M Begni B Galimberti G Rosanna G Ferrarese C Increased plasma glutamate in stroke patients might be linked to altered platelet release and uptake J Cereb Blood Flow Metab 2005 25 513 519 10.1038/sj.jcbfm.9600039 15660099

[B38] Kaufman RJ Orchestrating the unfolded protein response in health and disease J Clin Invest 2002 110 1389 1398 12438434 10.1172/JCI16886 PMC151822

[B39] Ferri KF Kroemer G Organelle-specific initiation of cell death pathways Nat Cell Biol 2001 3 E255 E263 10.1038/ncb1101-e255 11715037

[B40] Puthalakath H O’Reilly LA Gunn P Lee L Kelly PN Huntington ND Hughes PD Michalak EM McKimm-Breschkin J Motoyama N Gotoh T Akira S Bouillet P Strasser A ER stress triggers apoptosis by activating BH3-only protein Bim Cell 2007 129 z 1337 1349 10.1016/j.cell.2007.04.027 17604722

[B41] Nakagawa T Zhu H Morishima N Li E Xu J Yankner BA Yuan J Caspase-12 mediates endoplasmic-reticulum-specific apoptosis and cytotoxicity by amyloid-beta. Nature 2000 403 98 103 10.1038/47513 10638761

[B42] Idrissi EA Trenkner E Growth factors and taurine protect against excitotoxicity by stabilizing calcium homeostasis and energy metabolism J Neurosci 1999 19 9459 9468 10531449 10.1523/JNEUROSCI.19-21-09459.1999 PMC6782936

[B43] Bergamini L Mutani R Delsedime M Durelli L First clinical experience on the antiepileptic action of taurine. Eur Neurol 1974 11 261 269 10.1159/000114324 4212017

[B44] McCarty MF The reported clinical utility of taurine in ischemic disorders may reflect a down-regulation of neutrophil activation and adhesion Med Hypotheses 1999 53 290 299 10.1054/mehy.1998.0760 10608263

[B45] Schäbitz WR Developing granulocyte-colony stimulating factor for the treatment of stroke: Current status of clinical trials Stroke 2006 37 1654 10.1161/01.STR.0000227299.62106.0e 16728676

[B46] Aliprandi1 A Longoni M Stanzani L Tremolizzo L Vaccaro M Begni B Galimberti G Garofolo R Ferrarese C Increased plasma glutamate in stroke patients might be linked to altered platelet release and uptake J Cereb Blood Flow Metab 2005 25 513 519 10.1038/sj.jcbfm.9600039 15660099

[B47] Maragos WF Greenamyre JT Penney JB Jr Young AB Glutamate dysfunction in Alzheimer’s disease: an hypothesis Trends Neurosci 1987 10 65 68 10.1016/0166-2236(87)90025-7

[B48] Greenamyre JT Glutamate-dopamine interactions in the basal ganglia relationship to Parkinson’s disease J Neural Transm Gen Sect 1993 91 255 269 10.1007/BF01245235 8099800

[B49] Yu Z Luo H Fu W Mattson MP The endoplasmic reticulum stress-responsive protein GRP78 protects neurons against excitotoxicity and apoptosis: suppression of oxidative stress and stabilization of calcium homeostasis Exp. Neurol 1999 155 302 314 10.1006/exnr.1998.7002 10072306

[B50] Kitao Y Ozawa K Miyazaki M Tamatani M Kobayashi T Yanagi H Okabe M Ikawa M Yamashima T Stern DM Hori O Ogawa S Expression of the endoplasmic reticulum molecular chaperone (ORP150) rescues hippocampal neurons from glutamate toxicity J. Clin. Invest 2001 108 z 1439 1450 11714735 10.1172/JCI12978 PMC209417

[B51] Sokka AL Putkonen N Mudo G Pryazhnikov E Reijonen S Khiroug L Belluardo N Lindholm D Korhonen L Endoplasmic reticulum stress inhibition protects against excitotoxic neuronal injury in the rat brain J Neurosci 2007 27 901 908 10.1523/JNEUROSCI.4289-06.2007 17251432 PMC6672923

